# Understanding the roles of community health workers in improving perinatal health equity in rural Uttar Pradesh, India: a qualitative study

**DOI:** 10.1186/s12939-021-01406-5

**Published:** 2021-02-23

**Authors:** Andrea K. Blanchard, Shahnaz Ansari, Rajni Rajput, Tim Colbourn, Tanja A. J. Houweling, Shajy Isac, John Anthony, Audrey Prost

**Affiliations:** 1 Institute for Global Public Health, University of Manitoba, R070 Medical Rehabilitation Building, 771 McDermot Avenue, Winnipeg, R3E 0T6 Canada; 2 India Health Action Trust, 404, 4th Floor Ratan Square, Vidhan Sabha Marg, Lucknow, 226001 India; 3 Institute for Global Health, University College London, 30 Guilford Street, London, WC1N 1EH UK; 4 Department of Public Health, Erasmus MC, Dr. Molewaterplein 40, 3015 GD Rotterdam, Netherlands

**Keywords:** Maternal and newborn health, Community health workers, Health equity, Qualitative research, India

## Abstract

**Background:**

Despite substantial reductions in perinatal deaths (stillbirths and early neonatal deaths), India’s perinatal mortality rates remain high, both nationally and in individual states. Rates are highest among disadvantaged socio-economic groups. To address this, India’s National Health Mission has trained community health workers called Accredited Social Health Activists (ASHAs) to counsel and support women by visiting them at home before and after childbirth. We conducted a qualitative study to explore the roles of ASHAs’ home visits in improving equity in perinatal health between socio-economic position groups in rural Uttar Pradesh (UP), India.

**Methods:**

We conducted social mapping in four villages of two districts in UP, followed by three focus group discussions in each village (12 in total) with ASHAs and women who had recently given birth belonging to ‘higher’ and ‘lower’ socio-economic position groups (*n* = 134 participants). We analysed the data in NVivo and Dedoose using a thematic framework approach.

**Results:**

Home visits enabled ASHAs to build trusting relationships with women, offer information about health services, schemes and preventive care, and provide practical support for accessing maternity care. This helped many women and families prepare for birth and motivated them to deliver in health facilities. In particular, ASHAs encouraged women who were poorer, less educated or from lower caste groups to give birth in public Community Health Centres (CHCs). However, women who gave birth at CHCs often experienced insufficient emergency obstetric care, mistreatment from staff, indirect costs, lack of medicines, and referrals to higher-level facilities when complications occurred. Referrals often led to delays and higher fees that placed the greatest burden on families who were considered of lower socio-economic position or living in remote areas, and increased their risk of experiencing perinatal loss.

**Conclusions:**

The study found that ASHAs built relationships, counselled and supported many pregnant women of lower socio-economic positions. Ongoing inequities in health facility births and perinatal mortality were perpetuated by overlapping contextual issues beyond the ASHAs’ purview. Supporting ASHAs’ integration with community organisations and health system strategies more broadly is needed to address these issues and optimise pathways between equity in intervention coverage, processes and perinatal health outcomes.

**Supplementary Information:**

The online version contains supplementary material available at 10.1186/s12939-021-01406-5.

## Background

Despite experiencing large declines in maternal and neonatal mortality over the past decade, India accounts for the largest proportion of maternal and perinatal deaths in South Asia [1]. In 2015–16, the state of Uttar Pradesh (UP) had the highest estimated perinatal mortality rate in India, at 56 per 1000 births [2]. This is in part due to low and inequitable uptake of services for maternal and newborn health [3, 4]. Perinatal mortality rates are also disproportionately high among those who are socio-economically worse off [4].

Community Health Worker (CHWs) programmes have been implemented in numerous countries to improve Reproductive, Maternal, Newborn and Child Health (RMNCH). CHWs are trained to work in their own communities and are meant to be accountable to them [5–7]. There is considerable evidence on the positive effects of CHW interventions on RMNCH outcomes including institutional delivery, essential newborn care (ENC), danger sign recognition and newborn referrals in low- and middle-income countries (LMICs) including India; these interventions have contributed to reducing mortality during the perinatal period [8–15].

The Government of India’s National Rural Health Mission (NRHM), renamed the National Health Mission (NHM), was launched in 2005 aiming to improve RMNCH and other priority areas of public health [16]. The programme recruited and trained a cadre of CHWs called Accredited Social Health Activists (ASHAs). ASHAs are trained and incentivised to visit all pregnant women and new mothers at home, with specific guidance on how to reach those who are most socially or geographically marginalised [16]. In 2014, the India Newborn Action Plan was developed with ‘equity’ in newborn health as one of its guiding principles [17]. The plan described how ASHA home visits could strengthen equity by helping women from disadvantaged families to prepare for birth and deliver in a health facility, as well as reach them with advice on essential newborn care.

Research to understand the processes by which community-based programmes can improve socio-economic inequities in RMNCH is sparse. Some studies have examined the effects of community group or midwife interventions on equity in RMNCH outcomes in LMICs [18–21], but only a few have qualitatively explored the processes by which CHWs’ home visits may improve RMNCH overall in South Asia [22–24]. No studies have specifically explored whether ASHAs’ home visits can improve perinatal health outcomes equitably between socio-economic groups and how this is shaped by contextual factors. Therefore, we conducted qualitative research to explore how ASHAs’ home visits may or may not have improved equity in perinatal health between socio-economic position (SEP) groups in rural villages in Uttar Pradesh.

## Methods

### Study design

Since 2013, the state of UP’s branch of the NHM has been supported by the Uttar Pradesh Technical Support Unit (UP TSU). The UP TSU is funded by the Bill & Melinda Gates Foundation and implemented through a partnership between the India Health Action Trust (IHAT), the University of Manitoba, and other national and international organisations [25]. Our qualitative study was a component of a mixed-methods doctoral study led by the first author in collaboration with UP TSU, which combined distributional with relational analytical approaches to understand the extent to which, and processes by which, ASHAs’ home visits may improve perinatal health equity [26]. A distributional approach focuses on measuring differences or gradients in health outcomes between groups of people. Relational approaches derive from ‘practice theory’, which views human practices, including those related to health, as processes produced through social relations over time and space [27, 28].

The qualitative methods developed for this study were informed by Aziz et al.’s use of the relational concept of “social space” to explore the processes by which women are included or excluded by community-based RMNCH programmes within village contexts [29]. Our study considered socio-economic position in terms of interrelated socio-economic characteristics that relatively positioned people with more or less resources, power and status through socio-spatial relations [30–32]. We also explored ASHAs’ roles in equitably improving perinatal health outcomes and how these were influenced by contextual factors. Our approach was informed by a systematic review that we conducted, which showed evidence that the effects of CHW interventions on equity in maternal and newborn health could be influenced by a combination of CHW characteristics and intervention strategies, as well as contextual issues including community processes and supports, health care system, and the political, socio-economic and geographical context [33–35].

### Study setting

In the NRHM programme, ASHAs were meant to be trained to undertake five main activities: home visits; attend the Village Health and Nutrition Day (VHND) that is led by the Auxiliary Nurse Midwives; visit the health facility with pregnant women, sick children, or others; attend monthly review meetings; hold village-level meetings as member or member secretary of the Village Health, Sanitation and Nutrition Committee (VHSNC); provide leadership for developing and carrying out the village health plan; and maintain records to organise and track their work. To improve perinatal health, ASHAs should visit women at least three times per trimester of pregnancy, and three times in the week after birth [36]. ASHAs were trained more recently to provide home-based newborn care and community-level curative care for minor illnesses [37, 38]. The intended programme pathway from ASHA home visits to perinatal health outcomes overall is shown in Supplementary Figure 1.

To address equity in coverage and behavioural outcomes of the ASHA programme, the NHM produced a small guidebook called “Reaching the Unreached” on how ASHAs should identify and visit socio-economically and geographically marginalised households, provide repeated counselling to change their behaviours and generate demand to access to services, provide community level care, mobilise and accompany them to use services, and anticipate geographic, cultural, caste and other social barriers to these efforts [39].

Uttar Pradesh (UP) is the largest and most populous state in India with notable diversity [2]. The village was considered a key site where socio-economic position inequities in health could be studied relationally, because of its geographic boundedness and because it is the unit in which the ASHA programme is implemented. We purposively selected four village sites, first by identifying two distinct districts: one with larger and one with smaller differences in perinatal mortality between caste and education groups, based on UP TSU monitoring data. Allahabad in the East and Rampur in the West were among the districts that reflected these two patterns respectively, and represent important geographic and socio-cultural differences. Within each district, we selected two villages having a maximum of 5000 people, multiple ASHAs, and socio-demographic diversity. Two villages were chosen per district, one in a block (sub-district) nearer and one further from the headquarters, to represent differential access to health and other services.

### Data collection tools and methods

Data was collected by two research investigators (S. Ansari and R. Rajput) from UP, and a qualitative researcher from Canada (A.K. Blanchard). The researchers were women with postgraduate degrees in population health and sociology, and experience working in rural UP and elsewhere in India. AKB lived in India for 6 years and subsequently worked there for 10 years, including 1 year based in Lucknow for this study. While this provided a deeper awareness of the cultural, socio-economic, political, and health-related contexts in north and south India, having a different socio-demographic, language and cultural background from the participants could have affected their openness or responses in data collection. The data reflected that women from a range of backgrounds agreed to participate, and they talked reasonably openly about both positive and challenging experiences. We hope this was in part because they could perceive that the researchers respected them and were interested in their experiences.

The research team first met with stakeholders in each village to gain permission to conduct the study in December 2016, then returned for social mapping in February 2017. The researchers visited each village with the UP TSU’s District Community Specialist (DCS), and talked with CHWs (ASHAs, ASHA *sanginis* or mentors, and *anganwadi* workers), and the *pradhan* (village chief) or other leaders. We visited each hamlet where different social groups lived to observe living conditions and speak to pregnant or new mothers and family members. We recorded the discussions as field notes. The research investigators drew a social map during the process, and finalised it with help from village members.

The researchers conducted focus group discussions (FGDs) in May 2017. We developed FGD screening tools based on the characteristics that people used to define lower and higher socio-economic positions during social mapping (the general tool is shown in Table 1). Local UP TSU staff and CHWs used the tool to purposively identify and invite participants in person.

**Table 1:** Screening tool for FGD participant recruitment

Socio-economic characteristics	Lower socio-economic position participants	Higher socio-economic position participants
**House materials**	Semi-*pukka*, semi-*kuccha*, or *kuccha*– made of mud or thatched ceiling, only partly cemented (floor and/or roof not cemented)	*Pukka* or semi-*pukka* – made of cement on roof and/or floor and walls
**Water/electricity/toilet facility**	Hand pump or not, but no electricity and no toilets	There is at least a hand pump and/or well and electricity available, maybe also toilets
**Occupation of family and related income**	Labour work in or outside of village, brick maker, farming on others’ land that they do not own	Office or government jobs, owns a good amount of land, or private owner of shops or trades
**Caste status**	Scheduled Caste or Tribe *jatis*, or others considered of lower socio-economic positions in that village	General or Other Backward Class *jatis*, or others considered of higher socio-economic position in that village
**Literacy and education level**	Cannot read or write, or had no schooling	Can read or write, or had some schooling
**Location in village**	Living in a hamlet or section far from the main area of village	Living in the main hamlet or central area of hamlet

We conducted 12 FGDs, with three groups in each of the four villages: one with women of lower socio-economic position, one with women of higher socio-economic position, and one with ASHAs. FGDs with women who had recently given birth occurred in locations that were seen as convenient and comfortable, including the public school, *anganwadi* center, or home of the *pradhan* or ASHA. All FGDs were held privately with participants, their infants if they did not have child care, and the three researchers. The FGDs with ASHAs were held in a private room at the CHCs nearest to the study villages.

The moderator (S. Ansari) and note-taker (R. Rajput) sought individual written informed consent through signature or thumbprint for participation and audio-recording. They verbally described the research aims and process, confidentiality and voluntary withdrawal, and intended uses for informing the programme, and asked if they had questions. While participants were given the option to withdraw consent at any time, it is possible that women could have felt pressure to stay. Some women left during the pilot tested FGD to attend other responsibilities, indicating they felt able to leave, while no participants in the final FGDs left early. The research investigators then asked for anonymous information on participants’ socio-demographic characteristics. The FGDs were guided by a semi-structured tool. It was initially developed in English and refined by translating into Hindi and back-translating to English, before pilot testing in another district. After pilot-testing, we found it worked best to ask what participants did or experienced, then any alternative experiences or practices they knew others did, and then probed for any reasons for these differences to aid comparisons. We added a couple probes on recurring topics including changes over time and seasonality of newborn illness or death. The FGD guide is in Supplementary Table 1.

The note-taker drew a diagram of where each participant sat, with numbers corresponding to their socio-demographic information, and she noted their number when each participant spoke. Afterwards, we debriefed with participants and shared refreshments. The FGDs lasted between 50 and 70 min. Member checking of results with the participants was not possible due to logistical constraints. The aggregated results were shared with the UP TSU District Community Specialist and their team to provide additional insights during our interpretation of results. We found patterns in experiences and views emerging across the FGDs in the four villages, indicating that we were getting to data saturation [40].

Ethics approval for all data collection tools, protocols, and analyses was obtained from the Sigma Institutional Review Board in New Delhi [#10040/IRB/D/16–17] [41], and the University College London Research Ethics Committee [#9909/001].

### Analysis

The FGD recordings were translated from Hindi directly into English transcripts by a professional translator from UP. The translator retained words in Hindi that were not directly translatable or had multiple meanings. The research investigators also transcribed recordings into Hindi separately, and corroborated their meaning with the translator’s transcripts. English transcripts were imported into NVivo 11 and Dedoose. We also imported the table of participants’ socio-demographic characteristics as case classification sheets and assigned each to a case node.

We analysed the results using a thematic framework approach, which is widely used for qualitative analyses within a mixed-methods approach [42]. A coding structure with family and child nodes was first developed deductively based on the research questions, and our systematic review on the potential programme and contextual factors to explore [35]. The codes covered the health outcomes under study, the characteristics of higher and lower socio-economic position groups and the spaces where they lived, community health worker characteristics and roles, and factors related to the community and family, health services and system, geographical, political, and socio-economic contexts. We agreed on child nodes to be added inductively for recurring sub-topics. The coding structure is shown in Supplementary Table 2. The research team coded the first three transcripts in tandem, and compared and adjusted codes to ensure consistent meaning, then coded the rest. We also coded the text using the appropriate case node when each participant spoke.

Guided by the coding structure, we used NVivo queries to identify text on which characteristics were used to define people as having relatively higher or lower socio-economic positions by hamlet and village. The researchers ran matrix queries of the nodes on ASHAs’ home visits by differences in health outcomes between socio-economic position groups for each FGD, and summarised the coded text with key quotes in matrix charts in Excel. Finally, we ran matrix queries for text on each health outcome by each broad contextual factor, and summarised them in a matrix chart for each village, comparing between and within FGDs by participants’ characteristics. Using the descriptive matrix charts, the researchers developed initial themes through interpretive analysis to bring the results together on how ASHAs’ home visits may have improved equity in health outcomes between socio-economic groups, and the ways that contextual issues combined to influence this. We discussed the themes with the UP TSU team and all co-authors, and incorporated their input to finalise the results.

## Results

In total, there were 134 FGD participants: 53 women from lower socio-economic position groups, 35 from higher socio-economic position groups, and 46 ASHAs. Most FGDs had between 8 and 12 participants, but two had 13 and 15 respectively (Table 2).

**Table 2:** Number of FGD participants in the four study villages

Village/Block, District	Participants from lower SEP groups (n)	Participants from higher SEP groups (n)	ASHAs (n)	Total
**Village/Block 1, Allahabad**	12	7	11	30
**Village/Block 2, Allahabad**	14	8	12	34
**Village/Block 3, Rampur**	12	8	10	30
**Village/Block 4, Rampur**	15	12	13	40
**Total**	53	35	46	134

The FGD participants’ characteristics are shown in Supplementary Table 3 for women who recently gave birth. The results showed that people in all four villages understood socio-economic position in terms of six main characteristics: wealth (income or land ownership), access to facilities (electricity, water, sanitation, house materials), occupation, education, caste and religion. These characteristics appeared to overlap differently in each village to form local constellations in which people were positioned relationally with unequal identity-based status and respective resources and power. In Allahabad, occupation and wealth appeared to have the strongest joint role in stratifying lower to higher socio-economic positions. People described those with the lowest socio-economic positions as labourers working on other people’s agricultural land or in seasonal industries with low wages. The smaller contingent of people working in higher-paying, “formal” sectors were said to have the highest socio-economic positions. This hierarchy of occupations and related incomes was in many ways aligned with stratification by caste, particularly in village 2. The majority of people in Scheduled Caste (SC) groups (including all *harijans*, also labelled ‘untouchable’), as well as Scheduled Tribe (ST) groups in village 2, often worked as informal or agricultural labourers on others’ land. Meanwhile, many families of Other Backward Class (OBC) *jatis* (denoting a sub-caste or kinship group) and almost all people considered to be of a General Caste (GC) *jati* worked in the formal sector. These groups also most often held elected positions in village leadership.

In Rampur, people discussed socio-economic position in terms of occupation and related wealth, how these aligned with caste and religion, and to a greater extent than in Allahabad, education levels. Many OBC Muslim families worked in agriculture, mainly on their own small sections of land. They often relied on their children to work on the fields rather than attend school past fifth or eighth standards. In the Muslim-majority village 4, many SC Hindu families (from the same *harijan jati* as those in Allahabad who were labourers) owned their own land and had facilities like electricity and cement houses. They had somewhat higher educational levels compared to the OBC or GC Muslim *jatis*¸ though they lived on the edge of the village. Therefore, some SC/ST Hindu groups considered to have low identity-based status were not always economically worse off in relation to OBC or GC Muslim groups. Still there were patterns of higher resources, based on their occupation, wealth and education, among the caste and religion-based identity groups considered of higher status (for instance, OBC and GC groups who were Hindu).

As shown in Supplementary Table 4, most ASHAs were Hindu. During social mapping, this was explained in part because they more often met the educational requirement of completing eight or more standards and had more mobility than Muslim women. ASHAs were most often from OBC, and to a lesser extent Scheduled or General Caste *jatis*. Most had been working for over 10 years, but a few started recently to fill a vacancy. The VHSNC was not active in three study villages, but met every 3 months in the third village to discuss issues related to malaria, sanitation and hygiene, and the Village Health and Nutrition Day (VHND). We were told that the VHND occurred almost weekly in each village, usually at the local school, *anganwadi* centre, or an ASHAs’ home, where CHWs gave antenatal check-ups and immunisations. The CHC was only a couple kilometres to village 1 and 3, and over 10 km from villages 2 and 4. CHCs generally had more equipment and drug supplies in the first and third block, because they were in peri-urban areas, but their delivery loads were relatedly increasing. The CHCs had an average of four to five nurses and one or two doctors, with fewer on night duty; we were told that deliveries were often attended by staff nurses. The public district health centres were located in the district headquarters, over 20 km from village 1 and 4, and over 50 km from villages 2 and 3. Small private health clinics were available near each village, while larger private facilities were located in the headquarters further away.

### Roles of ASHAs’ home visits in influencing socio-economic equity in perinatal health

Our results indicated that the main roles of ASHAs’ home visits in improving perinatal health equity were building trusting relationships with women and their families, communicating about health services, schemes, and MNH care practices, and providing practical support for accessing public health services.

#### Building relationships with women and families

Participants said that it was important that ASHAs had built relationships to expand coverage of their home visits over time. ASHAs in both districts reflected on how they had to develop trusting relationships with families across socio-economic position groups over the last decade. The results also indicated they were received most readily by families of lower socio-economic positions, who had more to benefit from free services and incentives. ASHAs said that some families initially distrusted their intentions and roles: “Participant (unknown number): Initially, they would taunt us saying that we have nothing else to do except sit at people’s doors or that we are only coming to meet them because we are being paid for it.” (ASHAs Village 4) Yet people increasingly respected them, even among communities from which they were socio-economically distant. A Hindu ASHA in village 1 in Allahabad explained:

P (unknown): We keep going and meeting on and off, so they follow what we say. In fact, if they see us on the way and if there’s any kind of a problem, even if it’s a mild fever, they call us to look at whoever is sick. P (8): They have almost started regarding us as doctors ourselves! […] *Didi* [sister], I used to sit and cry earlier wondering how I would ever get to do work in my area. Seriously, it is a Muslim dominant area and I would be puzzled about how I would pursue them to go to the hospital. But now the situation has become really good and the people have become cooperative too. (ASHAs Village 1)

In this way, differences between families’ and ASHAs’ caste or religion were often overcome through their efforts to build relationships over time. For example, Hindu ASHAs met with Muslim families in Rampur, and many ASHAs from OBC *jatis* in Allahabad were able to meet people of *jatis* considered both of lower and higher positions than themselves. Still ASHAs’ and families’ relationships were constrained where there were wide socio-economic divisions in the village. For example, ASHAs in Allahabad’s second block described higher caste and elderly women as least receptive to their visits, though their relationships had improved over time. They said that families from one SC *jati* considered of lowest status living in an isolated hamlet also did not listen to them or seek hospital services.

#### Counselling on birth preparedness and institutional delivery

ASHAs’ efforts to communicate information and counsel families, particularly on affordable care, incentives, and professional care in case of complications, appeared to contribute to greater birth preparedness and institutional delivery. This was more evident among women from lower compared to higher socio-economic position groups. ASHAs in Allahabad’s second block said they counselled women and their families to bring money or clothes to the facility, and arrange a vehicle to the CHC. Two women in the lower socio-economic position group in village 3 explained how the ASHA promoted institutional delivery at the CHC, especially in case of complications: “P (6): She gives us good information. She tells us to deliver our children at the hospital […] P (gap): She advises us to have an institutional delivery and explains that in case of complications, we will not be able to manage the situation on our own. A doctor at a hospital can at least help us out in such a case.” (Lower SEP Village 3) One Hindu woman from an SC *jati* who had no education described her trust in ASHAs’ advice on these schemes: “M: Why do you feel that the information given to you by the ASHA worker is useful for you? P (6): She gives information that pertains to the schemes run by the government. If these schemes were not useful, why would the government be running them? And that is what they tell us.” (Lower SEP Village 3) Conversely, women from richer and higher caste groups most often planned to have private hospital deliveries. For example, an ASHA and her supervisor were advising a woman from a *jati* considered to be of highest status to go to the CHC, but she refused the advice:

P (6): There’s a woman in my area who has been married for 10 years and only recently conceived her first child. Four or 5 months into her pregnancy, the ASHA *sangini* [supervisor] and I were talking to her about her preferred facility for delivery. She told me she will manage on her own and that I shouldn’t interfere into her matter. M: What caste did they belong to? P (6): They were [highest status *jati*]. So, she did not listen to me […] I did not try to force her into listening to me. (ASHAs Village 2)

Overall, ASHAs said that they had seen tangible changes over time related to their counselling:

P (4): The information that we give them is very important because for instance, if they do not eat properly, they may experience weakness, anaemia, swelling in their hands and feet. But if they eat healthy and nutritious food, they will stay strong and even their babies will not have any complications […] M: Anyone else? What changes have you seen? P (2): There have been many changes […] for instance in many cases of delivery at home, the babies would die. Now at the CHCs that doesn’t happen so much […] P (6): My becoming an ASHA worker has helped to reduce the deaths of mothers and babies. (ASHAs Village 1)

#### Counselling on essential newborn care

Our results suggested that ASHAs provided less consistent counselling to women on ENC practices during pregnancy, and that visits were less frequent during the postnatal period. Women in each village said ASHAs had counselled them about early and exclusive breastfeeding: “M: Do your family members say anything about what should be fed to the baby? P (11+8): In earlier times, they used to ask to feed some syrup. But now, they follow whatever the ASHA worker says.” (Lower SEP Village 4) Yet the advice of ASHAs was often combined with guidance from family members, as these women in Rampur discussed: “P (6): She [ASHA] asks you to feed the baby with the mother’s milk. When she is in the hospital, she asks you to feed the baby with your milk first. If the elders are around, they [the elders] ask to feed cow’s milk.” (Higher SEP Village 2) Some women who gave birth in private facilities reported being told by the health personnel to feed the baby with formula while recovering from caesarean sections. Relatedly, the DCS indicated that private hospitals sometimes received commission from companies for promoting their formula.

For clean cord care, most people said the umbilical cord was cut with a new blade and either tied with string or clamped at the facility, but there was a lack of consensus between ASHAs, families, and health care staff on whether to apply antiseptic medication. Delayed bathing for at least 24 h after birth was more consistent, due to its alignment with a widely practised ceremony called *chhathi* that occurred after three or six (or a multiple of six) days after birth. Many participants said that ASHAs’ recommended delaying for at least 6 days. For home deliveries, which were more common in Rampur, women in village 3 said there were two *dais* who usually bathed the baby immediately. Staff nurses at CHCs, family members, or ASHAs also variously instructed women to place the baby on the chest after birth, though it was not clear that this was always skin-to-skin contact.

#### Practical support for accessing health services

ASHAs also provided practical support to prepare pregnant women and their families to access public health services, particularly for women of lower socio-economic positions who preferred the CHCs or otherwise had less support. ASHAs said they played a supportive role in women’s lives: “M: When you go to a pregnant woman in a village, how is your relation with that woman? P (3): […] See, they are able to share any issue with us like a sister or friend. If they need any aid for family planning, they ask us. These are the things they can only share with us and no one else, which is why we are like friends or sisters with them.” (ASHAs Village 4) This was especially true for women in Rampur whose family members all worked outside in agricultural labour: “P (3): They [family] think well of the ASHA worker because more often than not, they are not available to take us to the health centres. They then ask the ASHA worker to accompany us on account of themselves being occupied with some work. P (6): The ASHA worker accompanies us when we go for the delivery, so that is a big help.” (Higher SEP Village 3) A woman who was from a Muslim OBC *jati* with no schooling in village 4 said that the ASHA helped them reach the facilities: “P (8): Even at the time of the delivery, if a vehicle is not available, she arranges for her own motorcycle to help the woman commute. If nothing else, she walks on foot with the patient to the hospital.” (Lower SEP Village 4) A woman from an SC group in village 1 in Allahabad said that the ASHA played an instrumental role in her use of a public health facility by accompanying her: “P (7): I used to go to the private hospital during my pregnancy. But when the ASHA worker told me about the facilities of the government hospital, she herself took me there.” (Higher SEP Village 1)

In contrast, families who chose to give birth at private facilities usually arranged their own transport and finances. Some of the wealthier families also lived in the city most of the time, and accessed services there. In the first block in Rampur, nearer to the main city, ASHAs said they provided less support to those who were well-off: “P (8): It happens that if a woman’s husband is earning and living in the city, she gets her vaccinations in the village but goes to the city for her delivery only to come back later.” (ASHAs Village 3)

### Contextual issues affecting ASHAs’ roles in improving equity in perinatal health outcomes

Our analysis also suggested that there were three interrelated contextual issues that most challenged the roles of ASHAs in improving equity in perinatal health outcomes in the study villages, including existing socio-economic inequalities, geographical clustering, infrastructure and remoteness, and the perceptions and experiences of MNH services.

#### Socio-economic inequalities

First, ASHAs’ counselling and relationships were sometimes undermined by existing socio-economic inequalities and how they differentially affected people’s ability to receive adequate MNH care. Women of lower socio-economic positions in all villages said they chose to deliver at the public CHCs based on ASHAs’ counselling on the affordable public health care and incentives. One ASHA in Allahabad explained how people took great consideration of the costs:

P (9): When one delivery happened properly at the CHC and they received some monetary incentive too, now almost everybody comes here for delivery […] P (4 + 2): See, their very first concern is money. Here, they are able to save some money, they don’t have to pay for transportation, people can accompany them here for support along with the ASHA worker. Additionally, they also get incentives for institutional delivery […] P (4): They come here [CHC] because of the benefits. (ASHAs Village 1)

However, people’s trust in ASHAs’ counselling to give birth at public facilities was diminished when they faced indirect costs and delayed incentive payments. Women in the third village said there were numerous out-of-pocket-payments to make at the CHC: “P (5): See, the facilities are available but medicines have to be bought from outside, the nurses ask for money for sweets. So a lot of money is spent in this way.” (Higher SEP Village 3) Indirect costs at the public facilities (CHCs or district hospitals) led many families to take loans with high interest, which was difficult for poorer families to pay off, as women in village 2 described: “P (7): If you are poor, arranging for money is always difficult. P (3): We always have to take loans for going to the hospital. P (9 + 1): If you are a poor family and have no job, you will have a lot of trouble in arranging for the money.” (Lower SEP Village 2) ASHAs in that block of Allahabad, where the CHC was less resourced and further away, explained how indirect costs also caused many richer families to opt for the more expensive private hospital:

P (gap): Women from prosperous families do not come to the public facilities for this very reason. They blame us for not fulfilling our responsibilities and for not providing the facilities that we talk about […] P (1): See, even the women start reprimanding us later because we tell them that the services are available free of cost for them but they experience the opposite in the hospitals. P (9): They also say that since they have to pay for the services anyway, why should they go to the government hospitals at all when they can get better services in the private hospital […] P (gap): Another problem is that the pregnant women do not receive their due compensation [incentives] for up to 5 months. Then they blame us for making false promises about the facilities and medicines etc. available at the government hospitals […] (ASHAs Village 2)

A Muslim woman of a GC *jati* and no education in village 4 explained that the indirect costs and inconsistent receipt of incentives at government facilities caused her to stay home for her last birth, despite her family’s preference for a facility birth. ASHAs and women of higher SEP in village 2 also stated that receipt of incentives was related to people’s ability to open a bank account, which was harder for those with lower wealth, caste or migrant status because of a lack of identification cards or permanent addresses.

Women with higher socio-economic positions in village 3 of Rampur said they preferred private hospitals because they could afford them, while poorer families had no choice but to opt for public facilities: “P (8): Private is better for those who have money but for the poor, government hospital is the best. How can a poor person afford Rupees 50,000?” (Higher SEP Village 3) Reliance on private facilities was related to one’s social status and wealth, according to ASHAs in Allahabad: “M: Could you give an example of an instance when people did not want to come to the CHC? P (gap): The kiln owner in my village never comes to the CHC, they always go to the private hospital. M: Why is that? P (8): They feel insulted in coming to the CHC because they believe that only poor people will go to a CHC.” (ASHA Village 1) The DCS also described how a woman from a General Caste group had told him that her family would not allow her to deliver at the CHC and preferred private facilities due to a higher perceived quality and status.

#### Geographic clustering, infrastructure and remoteness

ASHAs’ ability to reduce inequities in perinatal health were also affected by geographical clustering of some lower socio-economic groups in disparate hamlets, the remoteness of some villages, and environmental barriers and limited infrastructure to link them. Living in a remote hamlet or village disproportionately affected families of lower socio-economic positions that sought to access health facilities to give birth, and increased the risk of maternal and perinatal deaths. ASHAs in village 2, located further from the headquarters in Allahabad, explained that the preference for home delivery among women of lower socio-economic groups was due to low education and resources, combined with geographical isolation, among women from *harijan* and *adivasi* groups. Similarly, in village 3 of Rampur, ASHAs said some women of OBC and SC groups found it inconvenient to go to the hospital to give birth: “P (8): There are certain people who keep assuring us that they will come to the hospital for their delivery and still deliver at home. M: Who are such people? P (9): There are a few belonging to the [name of OBC *jati*] caste and some from very poor families because they want to avoid the inconvenience of going far, or they worry about care-taking of their children while they are gone.” (ASHAs Village 3)

Women in all villages described delays in the ambulance reaching them or the hospital, especially in the Rampur villages that had busy railway tracks and a river to cross during monsoon season respectively. Women in the fourth village said they had to be taken by a makeshift boat to reach the highway leading to the clinic. Lower socio-economic position groups more often faced challenges to accessing timely and quality care, by virtue of living in more remote hamlets (village 1), or in blocks where public health care was seen as having lower quality (village 2 and 4), as two women in village 2 discussed:

P (gap): When I was in labour pain, my mother went and called the ASHA worker and even the ambulance arrived on time. In case of my eldest son’s delivery, it had been very late so I went to a private hospital [smaller clinic] for my delivery […] P (7): By the time the ambulance came to my house, my baby had already been delivered. I had been in so much pain for a long time but the moment my baby was delivered, I heard the ambulance drive at my doorstep. (Lower SEP Village 2)

Women living in isolated hamlets or villages also experienced delays when referred for emergency care at public district or private hospitals during labour, due to insufficient emergency obstetric care facilities at CHCs where more women of lower socio-economic position initially chose to give birth. Yet richer families who arranged for private transport and finances went directly to private facilities. ASHAs working in village 2 of Allahabad and village 3 of Rampur also noted that wealthier families from the village who lived in or went to the city before the delivery could more readily access these hospitals.

#### Perceptions and experiences of MNH services

ASHAs’ roles in improving equity in perinatal health were further affected by the differences in perceived and experienced quality of public and private health services available to each village. Mistreatment of women and families by staff had lowered perceptions of the quality of care at CHCs. In village 4 in Rampur, where home births were more common than other villages, ASHAs said that women who were reprimanded by staff nurses for coming too early before labour preferred to have home births subsequently. Women of less wealthy SC and OBC *jatis* in village 3 of Rampur discussed variable treatment by the nurses: “P (5): During my first child’s birth, the nurse had hit me. When I was in pain, she slapped me once on my cheeks and then once on my abdomen. P (6): See, the hospital has its advantages as well as its flaws. If even after a long time, the baby is not coming out, the nurse starts being rude. P (4): They behaved well with me.” (Lower SEP Village 3) Though women generally said that behaviour was the same for richer and poorer women at public facilities, participants in all villages said staff’s treatment differed based on whether they could give money and thus disadvantaged poorer families, as ASHAs in the second village observed:

P (11): Those who have money, they pay up front but those who cannot manage it from anywhere, the nurses threaten not to fill their forms and quarrel with them. P (12): Some people from the prosperous families anyway pay them up to Rs. 1000 or even Rs. 1200 out of free will. With such patients and even their ASHA workers, the conduct of the nurse is very kind and helpful. But for poor women, their conduct towards both the ASHA workers as well as the patients is very rude. (ASHAs Village 2)

Women who delivered at the private hospital, a larger proportion being of higher socio-economic positions, also said that staff treated them well because they paid money. In Rampur, ASHAs explained the preference for private hospitals in a similar way: “P (8): People go to private hospitals thinking that even though they are expensive, the conduct at such places is always good. If they go to government hospitals, they are stalled from one person to another. People say that for a lot of money in the private hospital, they are able to get good services.” (ASHAs Village 4) Further, all groups said their families trusted private over public care for treating newborn illnesses or complications. The lack of emergency obstetric care at CHCs also disproportionately disadvantaged those of lower socio-economic positions who initially planned to deliver there and had to be referred at the last minute, causing delays that could be fatal. An ASHA in the first block of Allahabad described the experience of a *harijan* woman who had a stillbirth at a private clinic after being refused at the CHC and not wanting to go further to the district hospital:

P (7): I brought a woman for delivery here [CHC] on [date]. The staff nurse asked me if there were any test reports for the woman available. I told her about the blood test reports. She asked about the ultrasound but they did not have any, she belonged to a low caste [*harijan jati*]. The nurse informed me that she was not able to detect the baby’s heartbeat and that it would not be possible to deliver the baby here. I asked the woman if she would go to the Allahabad [district] hospital but she refused. I took her to a local hospital [private] nearby but we found out there that the baby had died in the womb. (ASHAs Village 1)

In these ways, socio-economic inequalities, geographical clustering, variable infrastructure and remoteness, and perceptions and experiences of MNH services were important interrelated issues affecting ASHAs’ influence on birth preparedness, institutional delivery, and ultimately perinatal mortality among women of lower compared to higher socio-economic position groups.

## Discussion

This study explored how ASHAs’ home visits may be improving equity in perinatal health outcomes between socio-economic position groups in four villages of eastern and western UP. The results suggested that ASHAs’ home visits allowed them to fulfil three main roles: building trusting relationships, offering information about health services, incentives and preventive care, and providing practical support particularly for women of lower socio-economic positions. This was found to contribute to improved birth preparedness and institutional delivery rates, particularly at public CHCS, among women who were poorer, less educated or considered of lower caste status. Persistent inequities in institutional delivery and perinatal mortality were perpetuated by overlapping issues playing out within and between communities and health facilities, and their wider societal and health system contexts, that were largely beyond the ASHAs’ control.

Related research has largely focused on health equity in terms of achieving equitable coverage of interventions on one hand, or the distribution of health outcomes on the other, while less attention has been given to the pathways linking these together [35]. This study’s results suggest that equity in health practices and outcomes were influenced both by equity in coverage of home visits and counselling from ASHAs between socio-economic position groups, and in relational processes and practices between actors in community and health services that were shaped within the wider health system and societal contexts. This health equity pathway resonates with egalitarian theories of justice, such as Sen’s realisation-based theory, which argues for the need to pursue equity in both process and outcome [43]:

[I]n so far as processes and procedural fairness have an inescapable relevance to social justice, we have to go beyond health achievement and the capability to achieve health […] For this reason, inequalities even in health care (and not just in health achievement) can also have relevance to social justice and to health equity […] Furthermore, an adequate engagement with health equity also requires that the considerations of health be integrated with broader issues of social justice and overall equity, paying adequate attention to the versatility of resources and the diverse reach and impact of different social arrangements [43] (pp.660–1, 665).

Others have argued that this also involves understanding and addressing multiple features of the social organisation that allow some groups to achieve better health than others [44].

This study found that equity in coverage of home visits and counselling was largely achieved, as ASHAs had effectively developed relationships with people of various socio-economic backgrounds in line with their mandate [39]. Coverage could be further expanded to ensure they reach women in the most marginalised *jatis* and higher socio-economic groups. The situation was somewhat different from a study in Pakistan in which lady health workers were all from lower status *biradaris* (kinship groups), and therefore had trouble overcoming the social distance between themselves and women in their area from high status *biradaris* [22]. ASHAs had also played a role in equitably improving behavioural outcomes like birth preparedness and institutional delivery; ENC was low and could be further improved across all socio-economic groups. Two other qualitative studies in UP and neighbouring states found similar roles for ASHAs in improving institutional delivery [23, 45]. One of these studies found that while ASHAs’ counselling and support to families helped them to have facility births with JSY incentives, this was countered when families experienced greater barriers and opportunity costs at hospitals such as indirect payments, poor treatment or access, and limited JSY remuneration [45]. Our findings further showed that geographical clustering of lower socio-economic families in distant hamlets, or villages in more remote areas with less infrastructure caused them to face greater delays and financial barriers in accessing private facilities nearby, or higher-level public services further off, which could increase inequities in perinatal mortality. Another study showed that CHCs in UP that were further from the district headquarters and those that were not First Referral Units had a declining number of specialists between 2002 and 2012 [46]. A global review of factors influencing health service utilisation asserts the need to understand how issues like poor road and other infrastructure, less communication and information in remote areas aggravate socio-economic or other disadvantages, beyond approximating this with a single measure like ‘distance to hospital’ [47].

Our study also indicated that while ASHAs’ counselling influenced women of lower more than higher socio-economic groups to have an institutional delivery, the health care processes and relations involved in having an ‘institutional delivery’ could be inequitable and unjust themselves, in ways that ASHAs had little power to address. Other studies in north India also found that poor treatment, health personnel shortages, indirect costs and delayed incentives dissuaded some women from giving birth at hospitals despite ASHAs’ advice [23, 45]. Bohren et al.’s global review of evidence on mistreatment in maternal health care facilities identified a number of intersecting domains in which women experienced mistreatment from individual to health system levels, including the role of widespread stigma and discrimination on the basis of ethnicity, religion, socio-economic position, age and health status [48].

In this study, the inequitable processes and relations between people in the community and health services, and how they were shaped by health system, geographical and socio-economic contextual issues, were difficult for ASHAs to address through their interpersonal roles alone. Moreover, these issues often countered the relationships and trust they had built and aligned them more with the health services than the community, rather than a bridge between them, as Scott and Shanker described:

Rather than acting as cultural mediators seeking to improve the fit between government services and community needs, community members see their ASHA promoting services that do not necessarily serve them. Once an ASHA’s advice to go to the clinic proves unsound due to the limited institutional support, she loses face in the community and people are less likely to trust her on other matters [49] (p.1610).

Other research has suggested that, to date, ASHAs have more often served as ‘link workers’ to improve access to health services than as ‘social activists’ [50–52]. ASHAs’ current roles could be supported in a few ways to more comprehensively address inequities rooted in relational processes and broader contextual issues. Within the community, ASHAs could be supported to work more closely with other CHWs and organisations such as the VHSNC and women’s groups where they could advocate for addressing issues that women of lower socio-economic groups they know face, such as unequal distribution of village resources or access to the VHND, and issues like indirect costs or lack of free transport for health services [39]. In UP, investment in community mobilisation through women’s groups had not yet been widely prioritised, and in most places the existing groups had low attendance [53]. The national *Ayushman Bharat* Programme launched in 2018 reiterated the need to leverage the VHSNC, *Mahila Arogya Samitis* (women’s collectives with the ASHA as member secretary), and self-help groups to improve accountability of the health system and address other social determinants of health [52, 54, 55]. This may also require stronger partnerships between community, civil society and government actors to enhance social accountability [52]. Since 2016, the NHM also encouraged states to train ASHA facilitators and ASHAs in leading Participatory, Learning and Action cycles with women’s groups to identify and solve problems that pregnant women and new mothers face [56]. ASHAs could also have a stronger voice in health facility settings during cluster meetings and AAA forums by incorporating a focus on raising and addressing barriers to equity facing the families they support. Reviews on the evidence on health committees have shown how they are also affected by power relations in communities, health facilities, health systems and society in different, interconnected ways [57–59]. Therefore, both community and health facility organisations within which ASHAs could be better integrated must continually consider what can be done to collectively pursue equity in programme processes as well as outcomes.

To address wider health system and societal contextual issues, equity-focused strategies within and beyond the health sector would also be needed to prioritise the issues that disadvantage families of lower socio-economic positions. Research and policies to achieve effective coverage should consider whether improvements in quality and respectful maternal health care processes, such as through the recent LaQshya initiative, are equitable between socio-economic groups and whether this contributes to equity in their health outcomes [60–62]. Some of these issues may also be addressed through the *Ayushman Bharat* programme’s insurance scheme that explicitly aims to provide financial protection for poorer families, and covers both public as well as empaneled private hospitals where referrals are to be made [63]. Broader socio-economic, policy and health system changes are needed to undergird the ASHAs’ roles in bridging community and health services, and thereby improve equity in perinatal outcomes more comprehensively.

There were some limitations to the study. It used discussion-based more than observational methods, and the former better capture what people said they did than what they actually did. It would have been valuable to conduct repeat in-depth interviews to explore participants’ individual experiences or views and changes over time. It would have been challenging to conduct confidential in-depth interviews in women’s homes, and UP TSU staff suggested that FGDs would be more feasible and acceptable. We used FGDs with women of lower and higher socio-economic positions separately to gain diverse perspectives on the community programme, comparing between socio-economic groups within and between villages and districts. Recruitment relied on ASHAs’ list of all women who were pregnant or recently gave birth. While ASHAs should regularly monitor and update this list, using this for sampling could have caused selection and social desirability bias if more women who knew the ASHA participated. Four to five women who were approached from higher socio-economic groups did not agree to participate, because they or their family members were not comfortable with them leaving home. Though it was not the focus of this study, we spent time at the CHCs and asking medical staff about their experiences working there to help interpret the FGD responses. Future mixed-methods research would be valuable to explore differences in public and private health services’ quality, availability, accessibility and acceptability in this setting. In terms of transferability, the ASHA programme is fairly similar across India, but their characteristics and the socio-economic and health systems contexts where they work would vary.

## Conclusion

The results of this qualitative study suggest that ASHAs have had a positive influence on birth preparedness and institutional delivery, particularly among women of lower socio-economic positions, by building relationships, counseling and providing practical support. They seemed to have less influence on ENC and care for newborn illnesses across groups. It would be valuable to support ASHAs through stronger linkages with community and health service organisations, while attuning wider social and health systems efforts to tackling the local challenges facing lower socio-economic position groups. In this way, ASHAs’ roles would be better integrated within broader strategies that could optimise the pathway between equity in intervention coverage, processes, and ultimately perinatal health outcomes.

## Supplementary Information

**Additional file 1: Supplementary Figure 1.** Intended programme pathway from ASHA home visits to perinatal health outcomes.

**Additional file 2: Supplementary Table 1.** Focus group discussion tools with women who recently gave birth and ASHAs.

**Additional file 3: Supplementary Table 2.** Final coding structure for qualitative analysis.

**Additional file 4: Supplementary Table 3.** Socio-demographic characteristics of the FGD participants (women who had recently given birth).

**Additional file 5: Supplementary Table 4.** Socio-demographic characteristics of the FGD participants (ASHAs).

## Data Availability

The data are not publicly available to prevent the potential to breach confidentiality, but anonymised results are available from the corresponding author on reasonable request.

## Abbreviations

ASHA: Accredited Social Health Activist
CHC: Community Health Centre
CHW: Community Health Worker
DCS: District Community Specialist
ENC: Essential Newborn Care
FGD: Focus Group Discussion
GC: General Caste
LMIC: Low- and Middle-Income Countries
NHM: National Health Mission
OBC: Other Backward Class
RMNCH: Reproductive, Maternal, Newborn and Child Health
SC: Scheduled Caste
SEP: Socio-Economic Position
ST: Scheduled Tribe
UP: Uttar Pradesh
UP TSU: Uttar Pradesh Technical Support Unit
VHSNC: Village, Health, Sanitation and Nutrition Committee
