# Supplementary material for: Understanding the roles of community health workers in improving perinatal health equity in rural Uttar Pradesh, India: a qualitative study
Source: Int J Equity Health. 2021 Feb 23;20:63. doi: 10.1186/s12939-021-01406-5 (PMC7901073; doi:10.1186/s12939-021-01406-5)
Supplement: Supplementary file 1 — Additional file 1: Supplementary Figure 1. Intended programme pathway from ASHA home visits to perinatal health outcomes. [file 12939_2021_1406_MOESM1_ESM.pdf]

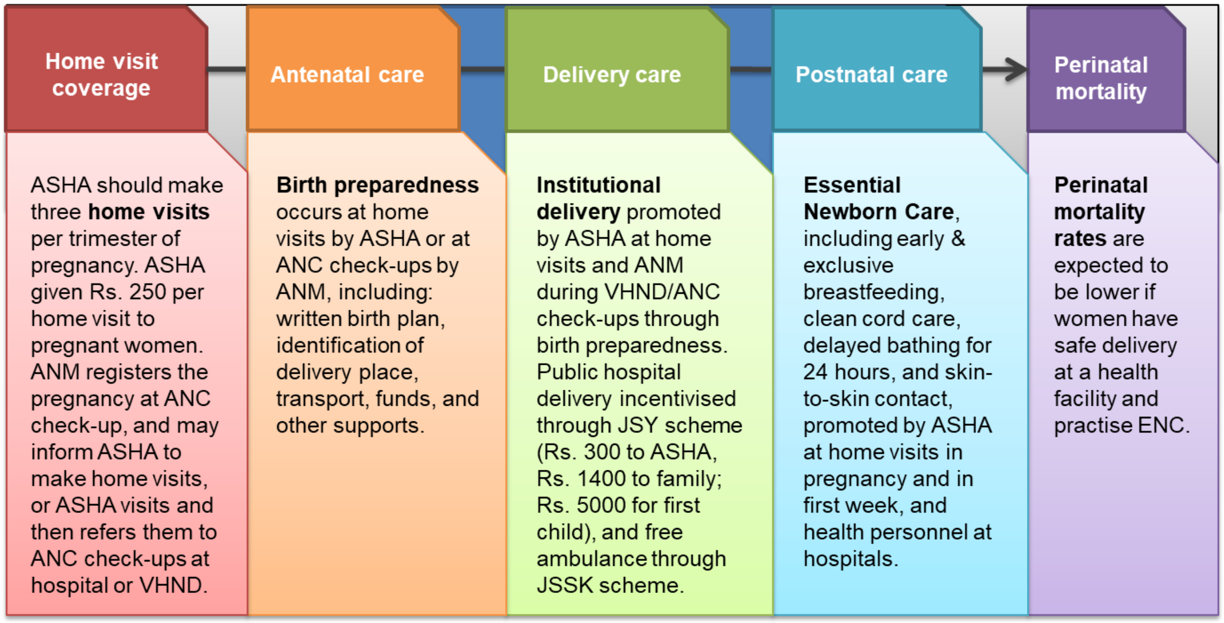

[ASHA: Accredited Social Health Activist; ANM: Auxiliary Nurse Midwife; ANC: Antenatal Care; VHND: Village Health and Nutrition Day; JSY: *Janani Suraksha Yojana*; JSSK: *Janani Shishu Suraksha Karyakram*; ENC: Essential Newborn Care]

Supplementary Figure 1: Intended programme pathway from ASHA home visits to perinatal health outcomes
