# Supplementary material for: Understanding the roles of community health workers in improving perinatal health equity in rural Uttar Pradesh, India: a qualitative study
Source: Int J Equity Health. 2021 Feb 23;20:63. doi: 10.1186/s12939-021-01406-5 (PMC7901073; doi:10.1186/s12939-021-01406-5)
Supplement: Supplementary file 2 — Additional file 2: Supplementary Table 1. Focus group discussion tools with women who recently gave birth and ASHAs. [file 12939_2021_1406_MOESM2_ESM.pdf]

Supplementary Table 1: Focus group discussion tools with women who recently gave birth and ASHAs

**Participant:** ***Women who recently gave birth***	**Focus Group Discussion Topic Guide 1 – English translation**
**Introduction**	*Thank you for coming today. As you know, we would like to know more about the home visits that Accredited Social Health Activists (ASHAs) are making in your area, the information they share on caring well for the woman and the new baby, and about going to hospital for delivery. Then we want to discuss for what socio-economic or other reasons women may or may not benefit from the advice that they share.*<br><br>First, we want to start by asking you: How long have you lived in this village? Did you deliver any children so far while living here before? How long ago was your delivery?
**Exercise 1: Social mapping of the village**	*Now we would like to discuss the map of this village created earlier, in answer to the following questions [for triangulation with the social mapping results]:*<br><br>- What are the outer boundaries of the community? Any landmarks to mark where it ends?<br>- Where are the different sections in the community (separate households/hamlets)?<br>- What are the main features of each section (hamlet) in the community?<br>o Roads, public transport stations, construction, drainage, garbage, water sources, electricity lines, phone lines<br>o Religious places, schools?<br>o Businesses, agricultural land, other livelihood activities (brick kilns, sand work)?<br>- Based on this, who has higher to lower well-being and socio-economic position? Why do you rank them this way? Probe: by caste, overall status, religion, house size/type, livelihoods, ownership of land/livestock, drainage and water, electricity, toilets etc.<br>- What maternal and newborn health problems exist here?<br>o How is the health status of mothers and newborns in each hamlet or section of the community?<br>- Where are the healthcare centers (*anganwadi* center etc.) and facilities (primary health centre, sub-centre, CHC), and their distances to different hamlets or sections within the community?<br>o Who among the people in different hamlets or sections participate in or accesses these facilities?<br>- Which community health workers work here and for how long?<br>o In which sections or hamlets of the community do they spend time or visit?<br>- Are there any social organizations like *gram panchayat*, self-help groups or VHSNC that meet in this community?<br>o Who is involved?<br>o Where do they meet?<br>o Are they involved in any of the maternal and newborn health activities here?
**Topic 1: Experience and opinions on access of health services**	*Now we would like to ask about your knowledge, experiences and opinions of the health services for pregnant and newly delivered ladies in your village.*<br>- Among the available health facilities, what services are provided there?<br>o Antenatal check-ups, immunizations, nurse visits? Primary/Community/District Health Centres, private, VHND? Who provides the services there?

**Topic 2: Experience and opinions of ASHA's activities**	**Views on ASHA's home visits in the community:** *Now we would like to ask about your views on ASHAs' home visits for pregnant ladies.*<br>- You have mentioned there are a few CHWs working here, like the ASHAs. What services do they provide?<br>o Did ASHA make a home visit to you? If so, how many times does the ASHA visit your home - Before delivery? In first week after delivery? For those who delivered in hospital, or home?<br>o Can you tell me about what the ASHA does in home visits for pregnant ladies? What kind of information do they share?<br>o What do you think about the work of ASHAs? How useful is the information that she gives?
**Topic 3: Differences in coverage, behaviours and outcomes, and related contextual factors**	*Now we can discuss if ASHA is making home visits and providing information, and for what reasons it is useful for women or not.*<br><br>***HOME VISITS***<br>- Do all people get home visits? Any women you know that ASHA did not visit during pregnancy (probe: who, what is their background?)<br>- Which family members are usually there during the home visit?<br>- What do family members think of ASHA's advice?<br>o Which groups (by hamlet, social background)? Why?<br>- Do ASHAs visit women who live in hamlets far from village?<br>- How are the roads, what areas are harder for her to get to?<br>- Are there any people who are not at home as often when ASHA comes to visit (like they go to farming or labour work)?<br>o What does the ASHA do if women are not living in the village? – any migrants, people of higher SEP living in the city?<br>- Is there anything that ASHA says that people find hard to understand or hard to follow? (probe: for what reasons?)
	DELIVERY PLACE<br>- Where did you give birth? What about other women you know? (probe re: different socio-economic positions)<br>- For what reasons you delivered there?<br>- Who in your family decides where to deliver? Where did you want to deliver?<br>- Did the ASHA influence you to go for a hospital delivery?<br>- Does the family take your ASHA's advice? (by caste, those who are not educated, poor?)<br>- If you delivered at hospital, how is the treatment by staff (probe: examples of those in a public hospital and those in a private facility)<br>- Those who delivered at hospital, what did you think about conditions of the facilities?<br>- For those with home delivery, what did you think about conditions during the home delivery (probe: who conducted and how?)<br>o For what reasons you thought it was good or not good?<br>- How do people get to the facility?<br>- Does anyone help you to go there (family, friend, ASHA?)<br>- What reasons it may be difficult to go (probe: living in far hamlets? Road conditions? Railway tracks, rivers?)<br>- If ambulance didn't come in time, what did you do?<br>- How do you find the cost of the hospital (probe: at public or at private? Any bribes taken by driver, staff?)<br>- What do you do to manage the costs? (probe: loans? any incentives (JSY)?)

	- Do people have support from *pradhan* [village head] or other community members for high-risk pregnancies?
	FEEDING the BABY<br>- How do you feed the baby after delivering? (probe: when to start breastfeeding, any other foods?)<br>- Did ASHA tell you anything about feeding the baby? If yes, what did she say?<br>- How do family members tell you to feed the baby?<br>- Who might be influencing you to breastfeed?<br>- What did the health personnel tell you about feeding your baby (probe: for those delivering in hospital? For those who had home delivery?)<br>- Any reasons it is hard for women to breastfeed? (probe: do they go for work, find it difficult? If so, what do they do?)
	CORD CARE<br>- What do you do with the cord after delivery? (probe: how is it cut? By whom? Did you apply anything?)<br>- What does ASHA tell you to do?<br>- Do others you know do different things to the cord? If so, what do they do? For what reasons?<br>- What do your family members tell to do with the cord?<br>- Did they take ASHA's advice or tell you something different?<br>- What do they tell women to do with the cord at the hospital? At home delivery? (for richer vs. poorer)?
	BATHING the BABY<br>- After delivery, when do you bathe the baby (after how many hours or days?)<br>- When did ASHA tell to bathe the baby?<br>- Do family members tell when to bathe the baby, and if so, when?<br>- Who tells you to do bathing in the hospital? For home delivery? (for richer vs. poorer)?<br>- Do you know others who do something different? For what reasons they do differently? Probe: Do they get water for bathing at home (for poorer and richer)?
	WARMING the BABY<br>- How do you keep the baby warm after delivery? (probe: do you wrap in something, put heat, or do skin to skin contact)? In first day? In first week?<br>- What did ASHA tell you about keeping the baby warm?<br>- What do family members tell you to do for warming the baby in the home? What do they tell?<br>- If so, did you listen to their advice or the advice of ASHA?<br>- Who influences you most on how to warm the baby- in the hospital? After home delivery?<br>- Do you know others who do something different, and if so what do they do? (Who? What social background?)<br>- For what reasons are they doing it differently?

	HEALTH of the BABY<br>- Do you know about any stillbirths or newborn deaths that occurred recently? At hospital or home delivery? (Probe: What was the background of that family?)<br>- What were the reasons the baby did not survive?<br>- Any newborn baby having illness you know of? (What was their background?)<br>- Did ASHA refer them to hospital?<br>- In that example, did the family go to hospital for that neonatal illness or death?<br>- If family went to hospital, how did they go there?<br>- Did anyone help them to take ambulance / own vehicle (Family, friend, ASHA?)<br>- What about if living in far hamlets? Any problems getting there? (road conditions, railway, rivers)?<br>- How did the doctor treat the newborn illness? Did they have the medicines or facilities needed?<br>- Did the family use the medicines?<br>- Any problem with the cost of medicines?<br>- Do people get afraid to give the medicines or injections?<br><br>- What is the most important thing that you can do to make sure the baby was healthy?
**Conclusion and debriefing**	*Now the main questions are finished. Thank you very much for sharing your time and experience. We would like to know what you thought about the discussion.*<br><br>**Debriefing:** How did you find the discussion? What did you like or not? What things did you feel were new or interesting? What did you feel were challenging, or did you feel uncomfortable or disagree? How will you remember this experience?
**Participant: *Accredited Social Health Activists***	**Focus Group Discussion Topic Guide 2 – English translation**
**Introduction**	*Thank you for coming today. As you know, we would like to know more about the home visits that Accredited Social Health Activists are making in your area, about the information ASHAs share on caring well for the woman and the new baby, and about going to hospital for delivery. Then we want to discuss the challenges that you face as ASHAs to go for home visits to some women and for what socio-economic or other reasons they are able to take your advice or not.*<br><br>First, we want to start by asking you each: How long have you worked in your village as ASHA? Why did you want to become an ASHA? How has your work changed over the years?<br><br>**Socio-demographic characteristics of their area:**<br>- What is the population of the area where you work?<br>- Are the households all in one area or very spread apart? How many hamlets are there?<br>- In your area, what are the characteristics of people by: caste, religion, assets, house type, livelihoods, educational level, ownership of land/livestock, available water/ electricity/ toilets?<br>- Based on these characteristics, who would you say has higher to lower well-being and socio-economic position? Why do you rank them this way?

**Topic 1: Experience and opinions on women's access to health services**	*Now we would like to ask about the health services for pregnant ladies in the village where you work.*<br>- Can you share what health services are provided where you work?<br>o Antenatal check-ups, nurse visits? Private, CHC/primary health centre/sub-centre, VHND? Who provides the services there?
**Topic 2: ASHAs' activities**	*Then we would like to ask you about the activities that CHWs do to help in the community.*<br>- Can you tell me about what services ASHAs provide during home visits?<br>o How do you identify women in a due list for making the home visits? Do you use any tools to help you plan your work? (probe: ASHA diary)<br>o If you are making a due list, is there some difficulty to visit some women on the list? If so who is it easier to visit more? Why or why not?<br>o Which parts of your work do you think are most important, and for what reasons?<br>o Which information do women find most important or useful?
**Topic 3: Differences in coverage, behaviours and outcomes, and related contextual factors**	*Now we can discuss if ASHA is making home visits and providing information, and for what reasons it is useful for women or not.*<br>***HOME VISITS***<br>- How is your relationship with women in your area?<br>- Do you find it difficult to visit some women? Who? What makes it difficult? (probe: by socio-economic background)<br>- Do any others help you to find pregnant women (ASHA *sangini*, *anganwadi* workers, auxiliary nurse midwives, or community people)?<br>- Which family members are usually in the home visit?<br>- What do the family members think about the information you gave?<br>- Any examples of when they did not accept your advice? What were the reasons? (probe: by hamlet, social background)?<br>- Any far away hamlets which are hard for you to reach often?<br>- How are the roads, what areas are harder to access?<br>- Are there any people who are not at home as often when you come to visit (like they go out for farming)?<br>- Are there any people who have a hard time to understand or follow what you tell them? What do you do about that?<br>- What do you do if people are not living in the village? Like any migrants, higher position live in the city?
	DELIVERY PLACE<br>- Where do people most often go for delivery? For what reasons do they go there?<br>- Does anyone not want to go for hospital delivery (public vs. private)? (probe: social background)?<br>- For what reasons they do not go?<br>- Do pregnant woman and/or their family decide where to deliver?<br>- Are there any cases where the family member did not take your advice? (Who? probe: caste, education, religion or wealth level)<br>- What did you do in that case?<br>- What do people think about the care at hospital (probe: primary/community/district health centre vs. private clinics)<br>- How is the behaviour of the staff there towards the people? Is it different for poorer people? Why?<br>- What do people think about the cleanliness and facilities in the hospital (probe: private, public)?<br>- How do people get to the institution? (probe: differences by social position)

	- Does anyone help them to take ambulance/ own vehicle (probe: family, friend, ASHA?)<br>- What about if living in remote hamlets? Road conditions?<br>- How do people find the cost? (probe: at private? public? extra costs?)<br>- What do people do to manage the costs, if any? (probe: loans, incentives) Is this a problem for people?<br>- Do people have support from *pradhan* or other community members for high-risk pregnancies?
	FEEDING the BABY<br>- How do women feed the baby after giving birth?<br>- Did you tell them anything about feeding the baby? If yes, what did you say?<br>- How do family members tell the woman to feed the baby? (probe: when to start breastfeeding, any other foods?)<br>- Are there any cases where the family member did not take your advice about feeding the baby? (Who? Probe: caste, education, religion or wealth level)<br>- What did you do in that case?<br>- What do they tell women to feed the baby if they delivered in hospital? For those who had home delivery? (family, ASHA, doctors/nurses)<br>- Any reasons it is hard for women to breastfeed? (probe: do they go for work, find it difficult?) If so, what do they do?
	CORD CARE<br>- What do people do with the cord after delivery? (probe: how is it cut? By whom? do they apply anything?)<br>- What do you tell them to do?<br>- Are there any examples of people who do different things to the cord?<br>- What do the woman's family members tell to do with the cord? (probe: how is it cut? By whom? Do you apply anything?) (probe: by socio-economic groups)<br>- Any examples of when they did not take your advice? What did they do instead? For what reasons?<br>- What do they tell women to do with the cord - at the hospital? At home delivery? (probe: by socio-economic groups)
	BATHING the BABY<br>- After delivery, when do people bathe the baby (after how many hours or days?)<br>- After how long after delivery do you tell them to bathe the baby?<br>- Do family members tell the women when to bathe the baby, and if so, when?<br>- Any examples of when they did not take your advice? What did they do instead? For what reasons?<br>- Who tells her when to bathe the baby - for hospital deliveries? For home delivery? What did they tell to do? (probe: by socio-economic groups)<br>- Any example of some women who did not bathe the baby at the right time? For what reasons?
	WARMING the BABY<br>- How do people keep the baby warm after delivery? (probe: do they wrap in something, do kangaroo mother care)? In the first day? In the first week?<br>- What do you tell women about keeping the baby warm?<br>- What do family members tell women to do for warming the baby in the home?<br>- Any examples of when they did not take your advice for wrapping or KMC? What did they do instead? For what reasons?

	- Who influences the woman most on how to warm the baby- in the hospital? After home delivery? What did they tell to do? (probe: by socio-economic groups)
	HEALTH of the BABY<br>- Any examples of stillbirths or newborn deaths you have observed or heard of? At hospital or home? (Where do those people live/ socio-economic background?)<br>- What were the reasons the baby did not survive?<br>- Any newborn baby having illness?<br>- Did you refer them to hospital? (probe: by socio-economic groups)<br>- In that example, did the family go to hospital for that neonatal illness?<br>- If family went to hospital, how did they go there? (probe: by socio-economic groups)<br>- Did anyone help them to take ambulance/ own vehicle (probe: family, friend, ASHA?)<br>- What about if living in far hamlets? Any problems getting there? (probe: road conditions, railway, river)<br>- How did doctor treat the newborn illness? Did they have the medicines or facilities needed?<br>- Did the family face problems with the cost of medicines? What did they do?<br>- Do people get afraid to take the medicines or injections?<br><br>- What are the most important things that one can do to make sure the baby is healthy?
**Conclusion and debriefing**	*Now the main questions are finished. Thank you very much for sharing your time and experience. We would like to know what you thought about the discussion.*<br><br>**Debriefing:** How did you find the discussion? What did you like or not? What things did you feel were new or interesting? What did you feel were challenging, or did you feel uncomfortable or disagree? How will you remember this experience?
