# Supplementary material for: Understanding the roles of community health workers in improving perinatal health equity in rural Uttar Pradesh, India: a qualitative study
Source: Int J Equity Health. 2021 Feb 23;20:63. doi: 10.1186/s12939-021-01406-5 (PMC7901073; doi:10.1186/s12939-021-01406-5)
Supplement: Supplementary file 3 — Additional file 3: Supplementary Table 2. Final coding structure for qualitative analysis. [file 12939_2021_1406_MOESM3_ESM.pdf]

Supplementary Table 2: Final coding structure for qualitative analysis

**Socio-economic position characteristics**
• Wealth or land ownership
• Religion
• Occupation
• Facilities in the village or home
• Education
• Caste
**Coverage and outcomes**
• Coverage of ASHA home visits
○ Antenatal home visits
○ Postnatal home visits
○ VHND, ANC or birth registration
○ Identification of pregnant women
○ Counselling by ASHA
○ ASHA diary
• Birth plan or preparedness
• Delivery place
○ Public hospital
○ Private hospital
○ Home
○ Informal doctor or midwife
○ Caesarean section
○ Any hospital
• Essential Newborn Care
○ Warming the baby
Skin-to-skin contact
○ Cord care
Cutting the cord
Anything applied to cord
○ Breastfeeding
Breastfeeding initiation
External food
Breast milk
○ Bathing of the baby
• Vaccination
• Birth outcomes and health
○ Stillbirth
○ Pregnancy complications
○ Newborn or infant illness
○ Neonatal, maternal, or infant mortality

**District, block, village, hamlets**
• Allahabad district
○ Village 1, Block 1 (+ hamlet child nodes)
○ Village 2, Block 2 (+ hamlet child nodes)
○ Rampur district
○ Village 3, Block 3 (+ hamlet child nodes)
○ Village 4, Block 4 (+ hamlet child nodes)
**Equity in coverage and outcomes**
○ Pro-equitable (favouring lower SEP)
○ Equitable (equal between SEP groups)
○ Inequitable (favouring higher SEP)
**Contextual factors**
• Community health worker characteristics
○ Relationship with women and family
○ Knowledge level
○ Coordination between CHWs
• Family dynamics and decision-making
• Community values, norms and supports
○ Traditional practices
○ Gender
• Health system and services context
○ Quality
Conditions of health facilities
Behaviour of health care workers
Perceived efficacy of treatment
○ Availability
○ Medicines or medical interventions
○ Affordability
Inexpensive
Indirect costs at public
Incentives
High costs at private
○ Accessibility
Own or private vehicle
Ambulance
○ Linkages and referrals
• Geographical, infrastructure, environment, or seasonal context
• Socio-economic context
• Political context
• Changes over time
