# Supplementary material for: Understanding the roles of community health workers in improving perinatal health equity in rural Uttar Pradesh, India: a qualitative study
Source: Int J Equity Health. 2021 Feb 23;20:63. doi: 10.1186/s12939-021-01406-5 (PMC7901073; doi:10.1186/s12939-021-01406-5)
Supplement: Supplementary file 4 — Additional file 4: Supplementary Table 3. Socio-demographic characteristics of the FGD participants (women who had recently given birth). [file 12939_2021_1406_MOESM4_ESM.pdf]

Supplementary Table 3: Socio-demographic characteristics of the FGD participants (women who had recently given birth)

District	Allahabad				Rampur			
Village/Block	Village/Block 1		Village/Block 2		Village/Block 3		Village/Block 4	
Participants	Lower SEP, n (%)	Higher SEP, n (%)[a.]	Lower SEP, n (%)	Higher SEP, n (%)	Lower SEP, n (%)	Higher SEP, n (%)	Lower SEP, n (%)	Higher SEP, n (%)
**Overall**	12	7	14	8	12	8	15	12
**Age group**								
18-22 years	9 (75)	3 (43)	5 (36)	3 (38)	7 (58)	4 (50)	6 (40)	2 (17)
23-27 years	2 (17)	4 (57)	8 (57)	5 (63)	4 (33)	4 (50)	6 (40)	7 (58)
28-35 years	1 (8)	0 (0)	1 (7)	0 (0)	1 (8)	0 (0)	3 (20)	2 + 1 over 35 (25)
**Religion**								
Hindu	12 (100)	7 (100)	14 (100)	8 (100)	12 (100)	8 (100)	4 (27)	3 (25)
Muslim	0 (0)	0 (0)	0 (0)	0 (0)	0 (0)	0 (0)	11 (73)	9 (75)
**Caste group**								
Scheduled Tribe (ST)	0 (0)	0 (0)	7 (50)	1 (13)	0	0 (0)	0 (0)	0 (0)
Scheduled Caste (SC)	6 (60)	5 (71)	2 (17) (1 *harijan*)	0 (0)	6 (50) (5 *harijan*)	0 (0)	4 (27) (all *harijan*)	3 (25) (all *harijan*)
Other Backward Class (OBC)	6 (50)	2 (29)	5 (42)	4 (40)	6 (50)	8 (100)	6 (40)	4 (33)
General Caste (GC)	0 (0)	0 (0)	0 (0)	3 (38)	0 (0)	0 (0)	5 (33)	5 (42) (all Muslim)
**Education**								
None	6 (50)	2 (29)	7 (50)	0 (0)	8 (67)	2 (25)	11 (73)	7 (58)
Lower primary (1-5 standards)	2 (17)	3 (43)	1 (7)	3 (38)	0 (0)	1 (13)	3 (20)	2 (17)
Upper primary (6-10 standards)	0 (0)	0 (0)	2 (14)	0 (0)	2 (17)	0 (0)	0 (0)	0 (0)
Secondary (10-12 standards)	3 (25)	2 (29)	4 (29)	4 (40)	2 (17)	3 (38)	1 (7)	3 (25)
College or university	1 (8)	0 (0)	0 (0)	1 (13)	0 (0)	2 (25)	0 (0)	0 (0)
**Husband's or families' occupation**								
None	1 (8)	0 (0)	1 (7)	0 (0)	1 (8)	0 (0)	0 (0)	0 (0)
Daily wage labour	9 (75)	6 (86)	10 (71)	0 (0)	8 (67)	0 (0)	6 (40)	5 (42)
Agriculture (own land)	1 (8)	1 (14)	0 (0)	1 (13)	2 (17)	2 (25)	6 (40)	7 (58)
Private or government	0 (0)	0 (0)	3 (21)	7 (88)	1 (8)	6 (75)	3 (20)	1 (8)

District	Allahabad				Rampur			
Village/Block	Village/Block 1		Village/Block 2		Village/Block 3		Village/Block 4	
Participants	Lower SEP, n (%)	Higher SEP, n (%)[a.]	Lower SEP, n (%)	Higher SEP, n (%)	Lower SEP, n (%)	Higher SEP, n (%)	Lower SEP, n (%)	Higher SEP, n (%)
**Number of children**								
1	5 (42)	1 (14)	3 (21)	2 (25)	2 (17)	2 (25)	2 (13)	2 (17)
2-3	6 (50)	4 (57)	5 (36)	5 (63)	5 (42)	6 (75)	5 (33)	5 (42)
4+	1 (8)	2 (29)	6 (43)	1 (13)	5 (42)	0 (0)	8 (53)	5 (42)
**Place of last delivery**								
Home	0 (0)	1 (14)	3 (21)	0 (0)	5 (42)	2 (25)	4 (27)	2 (17)
Public facility	10 (83)	6 (86)	10 (71)	1 (13)	7 (58)	3 (38)	8 (53)	4 (33)
Private facility	2 (17)	0 (0)	1 (7)	7 (88)	0 (0)	3 (38)	3 (20)	6 (50)

a. Due to vacancies in the community team we relied on, we had difficulties recruiting women considered to be of distinctively higher SEP groups in the first higher SEP FGD.
