# Supplementary material for: Understanding the roles of community health workers in improving perinatal health equity in rural Uttar Pradesh, India: a qualitative study
Source: Int J Equity Health. 2021 Feb 23;20:63. doi: 10.1186/s12939-021-01406-5 (PMC7901073; doi:10.1186/s12939-021-01406-5)
Supplement: Supplementary file 5 — Additional file 5: Supplementary Table 4. Socio-demographic characteristics of the FGD participants (ASHAs). [file 12939_2021_1406_MOESM5_ESM.pdf]

Supplementary Table 4: Socio-demographic characteristics of the FGD participants (ASHAs)

District	Allahabad		Rampur	
**Village/Block**	**Village/Block 1**	**Village/Block 2**	**Village/Block 3**	**Village/Block 4**
**Participants**	**ASHAs, n (%)**	**ASHAs, n (%)**	**ASHAs, n (%)**	**ASHAs, n (%)**
**Overall**	11	12	10	13
**Age group**				
20-29 years	3 (27)	4 (33)	2 (20)	2 (15)
30-39 years	5 (46)	4 (33)	4 (40)	10 (77)
40+ years	3 (27)	4 (33)	4 (40)	1 (8)
**Religion**				
Hindu	9 (82)	12 (100)	9 (90)	13 (100)
Muslim	2 (18)	0 (0)	1 (10)	0 (0)
**Caste group**				
Scheduled Tribe (ST)	1 (9)	0 (0)	0 (0)	0 (0)
Scheduled Caste (SC)	1 (9)	0 (0)	1 (*harijan*) (10)	2 (both *harijan*) (15)
Other Backward Class (OBC)	7 (64)	6 (50)	9 (90)	8 (62)
General Caste (GC)	2 (18)	6 (50)	0 (0)	3 (23)
**Education**				
Upper primary and lower secondary (6-9 standards)	8 (73)	0 (0)	5 (50)	9 (70)
Higher secondary (10-12 standards)	2 (18)	5 (42)	3 (30)	2 (15)
College or university	1 (9)	7 (58)	2 (20)	2 (15)
**Husband's or families' occupation**				
None	1 (9)	4 (33)	1 (10)	0 (0)
Labour	5 (46)	0 (0)	0 (0)	4 (31)
Agriculture/farmer (own land)	1 (9)	6 (50)	7 (70)	8 (62)
Private or government job	4 (36)	2 (17)	2 (20)	1 (7)
**Number of children**				
1	2 (18)	1 (8)	1 (10)	2 (15)
2-3	7 (64)	9 (75)	6 (60)	4 (31)
4-5	2 (18)	2 (17)	3 (30)	7 (54)

**District**	**Allahabad**		**Rampur**	
**Village/Block**	**Village/Block 1**	**Village/Block 2**	**Village/Block 3**	**Village/Block 4**
**Participants**	**ASHAs, n (%)**	**ASHAs, n (%)**	**ASHAs, n (%)**	**ASHAs, n (%)**
**Duration as an ASHA worker**				
<1-5 years	1 (9)	1 (8)	0 (0)	4 (31)
6-10 years	3 (27)	2 (17)	1 (10)	3 (23)
10-12 years	7 (64)	9 (75)	9 (90)	6 (46)
